# Evaluation of association between methylenetetrahydrofolate reductase and azoospermia: A meta-analysis: Retraction

**DOI:** 10.1097/MD.0000000000026037

**Published:** 2021-05-21

The article, “Evaluation of association between methylenetetrahydrofolate reductase and azoospermia: A meta-analysis”,^[1]^ which published in Volume 100, Issue 15 of *Medicine* is being retracted. The authors requested a retraction due to errors in the literature search. Authors mistakenly excluded studies that met the inclusion criteria, thus affecting the data analysis and validity of the results.
